# The Cyclopædia of Anatomy and Physiology

**Published:** 1835-07-01

**Authors:** 


*The Cyclopædia of Anatomy and Physiology*. Edited by Robert B. Todd, m.d., Lecturer on Anatomy and Physiology at the Westminster School of Medicine, &c. Part I.—London, 1835. 8vo. pp. 112.

We congratulate our readers on the appearance of the first part of this Cyclopædia. The following list of the articles contained in it will show how interesting are the subjects, and how capable are the contributors of elucidating them.

"Abdomen, by Dr. Todd; Absorption, Dr. Bostock; Acalephæ, Dr. Coldstream; Acids, Animal, W. T. Brande, Esq.; Acrita, R. Owen, Esq.; Adhesion, B. Phillips, Esq.; Adipocere, W. T. Brande, Esq.; Adipose Tissue, Dr. Craigie; Age, Dr. Symonds; Albino, Dr. Bostock; Albumen, W. T. Brande, Esq.; Amphibia, T. Bell, Esq.; Animal Kingdom, Dr. Grant."

The completion of the last article is reserved for the second Number, which is also to comprise the articles Animal, Ankle, Annelida, Anus, Aorta, Arachnida, Arm, Artery, and Articulation.

A few extracts may serve to give some notion of the execution of the work.

"Some curious anomalies have been observed in the venous circulation of the anterior abdominal wall, which, as being calculated to interfere with the operator, the practitioner would do well to note. M. Meniere has described a case in which a very large vein, arising from the external iliac, passed up along the linea alba to the umbilicus, was continued along the obliterated umbilical vein, and opened into the vena portæ. In another case, recorded by Manec, the vein originated in the same manner by two roots, reached the umbilicus, taking a course parallel to the umbilical artery, formed an arch outside the navel, and, having re-entered the abdomen, opened into the vena portæ. In another instance which occurred to Cruveilheir, the superficial veins in the hypogastric region were enormously enlarged; at the umbilicus they ended in a trunk as large as a finger, which communicated with the vena cava as it passed under the liver. Berard proposes to explain, by the supposition of the existence of such anomalies as those above described, the occurrence of fatal hemorrhages from wounds inflicted at the umbilicus, which have been attributed to the persistence of the umbilical vein." (Art. *Abdomen.*)

"*Cutaneous Absorption.* There is a branch of the subject to which we must now direct our inquiry,—the existence and extent of what has been deemed cutaneous absorption. When we trace the progress of the lymphatic vessels from their great central trunks, and follow them through all their minute ramifications, we find that many of them appear to have their origin from the surface of the body;* and hence we are led to suppose that the function of absorption is exercised, to a certain extent, by the cutis, or the parts immediately connected with it. That this is the case is proved by various pathological facts: we have daily opportunities of observing that various medicinal substances, by mere application to the surface, and still more when aided by friction, produce the same effect upon the system as if they had been received, in the ordinary way, through the medium of the stomach. By this

* See Haase, De Vas. Cut. et Intest. Absorb., tab. fig. 2; also, Mascagni, tab. 2, fig. 9—28, tab. 3.

means mercury manifests its specific action on the salivary glands; the salts of lead destroy the contractility of the muscular fibre; while opium, tobacco, and other narcotics, produce their peculiar effects on the nervous system.

"But, besides this kind of absorption, which is brought about by the substances being, as it were, mechanically forced into the pores of the skin, and thus applied to the mouths of the lymphatics, it was an opinion very generally embraced by the older physiologists, and still retained by many of our contemporaries, that the lymphatics, which are distributed over the surface, possess the power of imbibing water, when simply applied to it by the immersion of the body, or even when it is exposed to aqueous vapour diffused through the atmosphere. This supposed power of cutaneous absorption was called in to account for various physiological or pathological facts, for which it appeared to afford a plausible explanation; while, on the other hand, the easy mode in which it appeared to account for these facts was made use of as the great argument to prove its existence. The statical experiments of Sanctorius, which have, since his time, been so much multiplied and extended, were supposed to prove unequivocally that the body is capable of gaining weight, independently of any substance received into the stomach; and, to account for this addition, recourse was always had to the cutaneous absorption. Of late, indeed, it has been discovered, that a part of the effect ascribed by Sanctorius to the action of the skin is in reality due to the lungs; but still, after making the necessary deduction for the operation of the latter organ, there remained a certain increase of weight, which it was supposed could only be accounted for by admitting the existence of the cutaneous absorption.*

"The doctrine of cutaneous absorption has, however, been altogether called in question by Seguin, who performed a series of experiments, which consisted in immersing a part of the body in a saline solution; for example, that of corrosive sublimate, the effects of which on the system at large would be easily recognized, if any part had been absorbed. The result was, that, when the cuticle was entire, no effect, that could be attributed to absorption, took place; and the conclusion seemed not unnatural, that, under ordinary circumstances, it did not exist.† Currie was led to form the same conclusion by accurately weighing the body before and after immersion in the warm bath, under circumstances which were conceived to be favourable to the process;‡ and, as the results of his experiments coincided with those of Seguin and others, the doctrine of cutaneous absorption, except under the particular circumstances mentioned above, was very generally abandoned. Expe-

* "Mascagni, p. 22-3; see also Kellie, in Ed. Med. Journ., vol. i. p. 170 et seq.; and the article 'Integuments' in Rees's Cyclop.

† "Fourcroy, Méd. Eclair. t. iii. p. 232—241, and Ann. Chim. t. xc. p. 185 et seq.

‡ "Med. Reports, ch. xix.

riments have been adduced to prove that, even under these particular circumstances, when substances are applied by friction to the surface, they do not enter into the mouths of the vessels, but, being volatilized by the heat of the body, that the vapour thus produced is inhaled by the lungs;* an opinion which one might be inclined to think was almost too extravagant to be seriously maintained.

"The subject of cutaneous absorption has been lately investigated by Dr. Edwards, with that skill and address which he has applied to so many departments of physiology. By a number of experiments, which were performed on cold-blooded animals, where it was more easy to observe the effects, he found that absorption was carried on, to a considerable extent, when the animal, or a part of it, was immersed in water. The conclusion which the experiments seemed to warrant was, that transudation and absorption are, at all times, going forwards at the surface, but that the operations proceed at different rates, according to the circumstances in which the animal is placed, and that the body gains or loses weight, in proportion to the excess of one of them above the other. The analogy of the cold-blooded animals he applies to those with warm blood, and he supposes that they are subject to the same double action; a conclusion which appears to be confirmed by some experiments that were performed on guinea-pigs immersed in moist air, when an increase of weight was found to have taken place; which, after taking every circumstance into consideration, seemed necessarily to depend on absorption.† With respect to the experiments of Seguin, Dr. Edwards is not disposed to call their accuracy in question, but he points out various circumstances connected with them, which he conceives would tend to increase the transudation, and to diminish, or even entirely to suspend, the absorption.‡ The experiments of Dr. Edwards, considered in all their relations, are generally conceived to decide the question respecting the existence of cutaneous absorption, under the ordinary circumstances, and in the natural conditions of the system." (Art. *Absorption.)*

"*Secretion.* The existence of this function in the acalephæ is made known to us by the emission from their bodies, under certain

* "Ed. Med. Journ., v. ii. p. 10 et seq."

† "De l'Influence des Agens, &c., ch. xii. p. 345 et seq."

‡ "De l'Influence, &c. ch. xiii. p. 556 et seq. See, on this subject, Magendie, Physiol., t. ii. p. 189—196, and Dict. de Méd. et Chir. Prat. 'Absorption;' where he endeavours to prove that it is the veins, and not the lymphatics, which are the agents in cutaneous absorption. See also the remarks of M. Rullier, ch. ii.; and of M. Adelon, Physiol. t. iii. p. 10 et seq.; also art. 'Absorption,' Dict. de Méd. t. i. p. 124 et seq. M. Chaussier found that sulphuretted hydrogen gas, when applied to the surface of the body, manifested its deleterious effects on the system, Bibl. Méd. t. i. We have already had occasion to notice the opinion of Walter on this subject, p. 25, which is similar to that of M. Magendie. M. Buisson attempts to establish a distinction between the absorption which is carried on by the membranes and by the cellular texture, De la Divis. des Physiol. Phenom. p. 251 et seq."

circumstances, of a glairy mucus; by the stinging effect which some unknown product of their organization has upon our skin; and by the remarkable phenomenon of luminousness, which a large number of them present. The organs by which the mucus is secreted have not been satisfactorily observed. Dr. Milne Edwards saw reason to conclude, with regard to the rhizostoma, that a large quantity of this fluid is secreted by a glandular structure situated along the margins of the arms. The stinging property possessed by several animals of this class has been the subject of inquiry since the time of Aristotle; but to this day we remain in doubt with regard to the nature and mode of production of the agent which causes this effect. Some men seem to be insensible to the irritation generally produced by the contact of living acalephæ. But, for the most part, a slight touch of any part of their surface, and chiefly of the pendent tentacula, is followed, within a few minutes at most, by a burning pain, redness, swelling, and sometimes even a vesication, of all that portion of the skin which touched the animal. Sloane said of the physalus, ('what the seamen call caravels, or Portuguese men-of-war,) "They burn violently; they do suck themselves so close to the skin, that they raise blisters, and cause sometimes St. Anthony's fire."* Even on our own coasts, severe cases of inflammation of the skin are occasionally seen, which have been produced by the irritation received during bathing from some of the larger pulmograda." (Art. *Acalephæ.)*

We see, from Dr. Symonds' well-written essay on Age, that we were anticipated by no less a person than Lord Bacon in the interpretation of Medea's cauldron, which we gave in our review of Dr. Green's book. "Lord Bacon, in his curious and highly interesting treatise, entitled Historia Vitæ et Mortis, has much to say upon desiccation, and the methods of preventing it, such as bathing and inunction. The fable of the restitution of old Æsop [Æson] by the cauldron of Medea, he considers typical of the utility of the warm bath in softening the substance of the body." (P. 81.)

This first part does great credit to the talents of its distinguished editor, and the work, when completed, will be a library rather than a book. It is illustrated by an engraving, and a number of good woodcuts.

* "Nat. Hist. of Jamaica, ii. p. 273. Sloane recommends acajou oil as '*the* remedy for the stinging of this nettle.' Mr. Bennet has lately found (Lond. Med. Gaz. xiv. 908,) that the application of vinegar to the irritated surface in some degree alleviates the pain."

